# Healing of Recurrent Aphthous Stomatitis by Non-Thermal Plasma: Pilot Study

**DOI:** 10.3390/biomedicines11010167

**Published:** 2023-01-09

**Authors:** Norma Guadalupe Ibáñez-Mancera, Régulo López-Callejas, Víctor Hugo Toral-Rizo, Benjamín Gonzalo Rodríguez-Méndez, Edith Lara-Carrillo, Rosendo Peña-Eguiluz, Regiane Cristina do Amaral, Antonio Mercado-Cabrera, Raúl Valencia-Alvarado

**Affiliations:** 1 Interdisciplinary Center for Health Sciences CICS-UST, Instituto Politécnico Nacional, Av. Luis Enrique Erro S/N, Unidad Profesional Adolfo López Mateos, Zacatenco 07738, Mexico; 2 Plasma Physics Laboratory, Instituto Nacional de Investigaciones Nucleares, Carretera México Toluca S/N, Ocoyoacac 52750, Mexico; 3 Orocenter Clinic, Facultad de Odontología, Universidad Autónoma del Estado de México, Av. Paseo Tollocan esq. Jesús Carranza, Colonia Universidad, Toluca de Lerdo 50130, Mexico; 4 Departamento de Odontología, Universidade Federal de Sergipe, Aracajú 49060-108, SE, Brazil

**Keywords:** healing therapy, recurrent aphthous stomatitis, non-thermal plasma, recurrent ulcers

## Abstract

Recurrent aphthous stomatitis (RAS) is a common disease in the oral cavity characterized by recurrent ulcers (RU). Usually, these cause acute pain without definitive treatment. The present study determines the efficacy of non-thermal plasma (NTP) for treating RU. NTP is applied to the patient’s RU using a radiofrequency generator connected to a point reactor. The power density applied to the ulcer is 0.50 W/cm^2^, less than 4 W/cm^2^, which is the maximum value without biological risk. Each patient received two treatments of three minutes each and spaced 60 min apart at a distance of 5 mm from the RU. From a sample of 30 ulcers in patients treated for RU with an average age of 37 years, they stated that the pain decreased considerably and without the need for ingestion of analgesics and antibiotics. Regeneration took place in an average of three days. The NTP proved to be an excellent therapeutic alternative for the treatment of RU since it has a rapid effect of reducing pain and inflammation, as well as adequate tissue regeneration.

## 1. Introduction

**Figure 1:**
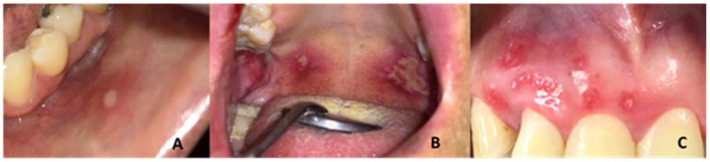
Clinical characteristics of: (**A**) minor RAS, (**B**) major RAS, (**C**) herpetiform RAS.

Recurrent aphthous stomatitis (RAS) is a disease that affects approximately 20% of the population, although the impact varies according to ethnic group and socioeconomic status [1,2]. It is characterized by lesions with a loss of continuity of the epithelium that manifest repeatedly, recurrent ulcers (RU), which are representative due to their frequency and symptomatology, constituting 20% of stomatological emergencies [3]. RAS is not a deadly disease but affects the patient’s quality of life. The lesions observed clinically are round or oval ulcers on the buccal mucosa and are covered by a pale or yellowish membrane with an erythematous outline. According to the size of the ulcers, they are classified into three types (Figure 1): minor RAS is the most common type where there are 1 to 5 ulcers less than one centimeter in diameter and they take seven to fourteen days to heal; major RAS, corresponds to 1 to 10 ulcers of more than one centimeter in diameter, which heal in up to thirty days; and herpetiform RAS corresponds to multiple ulcers (from 10 to 100) of 1 to 3 mm in diameter, which take between fifteen and thirty days to heal [1,4,5,6,7]. The RU compromises the mucosa of the lips, cheeks, background of the sack, oropharynx, tongue, the floor of the mouth, palate and gums [4,5].

The etiology of RAS remains unclear, so various factors have been proposed: immunological, hereditary, genetic, allergic, nutritional and microbial, as causative agents and there are no efficient curative treatments. Various treatments for RUs have been reported to heal these types of injuries or reduce pain [8,9,10,11]. Regarding pharmacological therapies prescribed, many medications include antiseptics, anti-inflammatories, antibiotics, corticosteroids, or thalidomide [2,12,13,14,15] and plant-based medicine [16]. In patients with constant and aggressive outbreaks (major canker sores), the pain is severe and typical treatment cannot provide symptom relief; therapy is indicated via corticosteroids (prednisone), among other drugs [15]. There are currently other alternatives, such as laser therapy [17,18,19,20,21], ozone therapy [22,23,24]; biological treatments such as Anti-TNF-alpha [6] and probiotics [25], which provide good results but are still need not an ideal alternative.

From the physical point of view, plasma is defined as the fourth state of matter. Depending on the thermal balance between electrons and atoms or gas molecules, it can be classified as a thermal plasma or non-thermal plasma [26]. Non-thermal plasma (NTP) can be generated by electrical discharges or gas bombardment with a high-energy electron beam at atmospheric pressure and, therefore, at room temperature [27]. In non-thermal plasmas, the temperature of ions, atoms and/or molecules, both excited and neutral, is close to room temperature. In contrast, the temperature of electrons is in the order of thousands of Kelvin. Thus, the electrons strengthen kinetic energy and the existence of the plasma through the formation of different energy states of heavy particles under the direct influence of the ionization process of some atoms and/or molecules present in the gas. In addition, high-temperature electrons can separate molecular gases such as oxygen and nitrogen, generating various chemical reactions. Chemical species in NTP include: the reactive oxygen species (ROS), such as atomic oxygen (O_2_), superoxide (O_2_^−^), ozone (O_3_), hydroxyl radical (OH) and hydrogen peroxide (H_2_O_2_); and the reactive nitrogen species (RNS), such as nitric oxide (NO), nitrogen dioxide (NO_2_), nitrogen trioxide (NO_3_), nitrous oxide (N_2_O), nitrogen oxide (N_2_O_4_) and positive ions such as N_2_^+^, of considerable importance in biomedicine [28,29,30,31,32].

Nowadays, application of NTP is used in biomedical procedures [33], such as disinfection [34], coagulation [35] and wound healing [36], among others. In particular, various research groups have reported the effects of reducing the healing time of wounds and ulcers. The present study applied NTP to RU to identify tissue repair time and symptomatology in patients with RAS.

## 2. Materials and Methods

Fourteen patients were selected who attended the Orocentro Clinic of the Dentistry Faculty from the Mexico State Autonomous University due to ulcers diagnosed as RU. The patients were over 18 years old, of both genders, without systemic compromise and agreed to participate in the study by signing an informed consent. Patients with oral soft tissue diseases, maxillary orthopedic, orthodontic treatment and those with maladjusted dental prostheses were excluded.

A longitudinal study was carried out to evaluate the tissue repair time of recurrent ulcers and the presence of pain. Once it was completed and the patient medical history had established the diagnosis of RU, data of size and location of ulcer were recorded, as well as the evaluation time and pain intensity. Pain intensity was measured using the 10-point visual analog scale (0 = “no pain at all”, 10 = “worst possible pain”) [37]. Once the patient had agreed to participate in the study and the informed consent was signed, the application of NTP with helium gas continued for three minutes at a distance of 5 mm, the ulcer was measured again and the intensity of the pain was assessed. After the first application, the NTP application was repeated under the same conditions (three minutes at a distance of 5 mm) and the ulcer was subsequently measured. Pain intensity was assessed similarly. The patients were evaluated every 24 h after the NTP application, with ulcer measurement and pain assessment, until tissue regeneration with surface continuity was identified.

The proposed atmospheric pressure NTP system is constituted by a radiofrequency (RF) power generator directly coupled to an *LC* resonant load, avoiding use of a matching network, where *L* is a non–linear inductor and *C* is the resultant capacitance of a coaxial cable connected to an ergonomic plasma reactor. The RF power generator is based on the 13.56 MHz class E RF module, which is supplied by three dc voltage sources, one of them providing an adjustable voltage magnitude represented by *V_DC_*. The available electric power at the RF module output terminals *P_RF_* is controlled by the magnitude of the applied dc voltage (*V_DC_*) [38].

**Figure 2:**
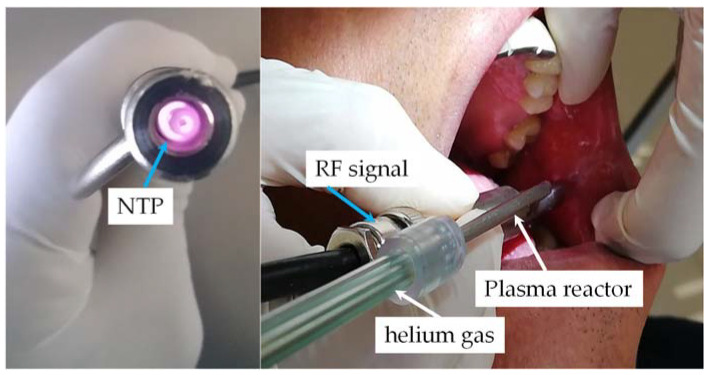
Plasma reactor and non-thermal plasma application in the patient.

For the NTP application generated with helium gas, the operating parameters reported in previous works were considered [34,36]. These are the radiofrequency (RF) and an electrical power generator of 20 W at a frequency of 13.56 MHz was applied to the plasma reactor, with which a power density applied to the ulcer is established based on the geometry of the reactor. The irradiance applied to the patient was 0.50 W/cm^2^, less than 4 W/cm^2^. With this irradiance, it is guaranteed that there is no biological risk, according to the data of the International Commission on Non-Ionizing Radiation Protection [39]. In the applications, 0.75 LPM of helium gas flows through the reactor and the exposure time in each of the two sessions per patient was 180 s. Once the plasma was generated, the tip of the reactor was kept at a distance of 5 mm from the ulcer (see Figure 2).

For the statistical analysis, descriptive and comparative studies were performed on ulcer type (Fisher’s exact test). Comparative analyzes (ANOVA one-way test, followed by Tukey) for pain and lesion size were also achieved.

## 3. Results

We measured the size of the ulcer and the erythematous contour in each case and took a photographic record. The sample consisted of 14 patients, of whom nine were women (64.3%) and five were men (35.7%). The age range was from 18 to 67 years old, with a mean of 37 years old. The patients presented from one to five ulcers, resulting in 30 ulcers. Of these, 19 (64%) were minor RU, 10 (33%) corresponded to major RU and 1 (3%) was herpetiform RU. The distribution of oral RU cases is shown in Table 1. The size of the ulcers ranged between 2 and 19 mm, with a mean of 7 mm. Before the application of the NTP, all patients reported pain on a numerical verbal scale between 0 and 10 (where 0 is no pain and 10 is the most intense pain); 75% of the patients reported pain, with a mean of 7.

When socioeconomic and risk factors are associated with the type of ulcer, a statistically significant association is verified for recurrence, alcohol use, medication use, occupation, education, marital status, age and gender (*p* < 0.05—Fisher’s exact) (see Table 1).

After performing the previous review in all cases after the second NTP application, six cases (20%) showed tissue regeneration in the ulcer, considering them repaired at zero days. In this exact measurement, 18 (60%) of the cases reported asymptomatic and the other 12 (40%) cases reported decreased pain with a mean of 4. At 24 h after the two applications with NTP, 11 cases (37%) showed total repair and the remaining 19 cases (63%) showed a reduction in the ulcer size of between 1 and 6 mm with a mean of 3 mm. Regarding the sensation of pain, when assessing the 30 cases, 27 (90%) reported asymptomatic, two cases (7%) reported pain with a value of 1 and the other case (3%) presented pain decreasing from a value from 7 to 4 on the visual analog scale. Ulcer repair time ranged from one hour to 7 days, with a mean of 3 days. In a single case, the ulcer persisted after applying NTP; in addition to the patient reporting pain, the patient reported positive for smoking. The time it took for the ulcers to regenerate showed a correlation only with the ulcer’s initial size of 0.703 (*p* = 0.000). Table 2 shows the evolution of the cases regarding ulcer size, pain and erythema size.

**Figure 3:**
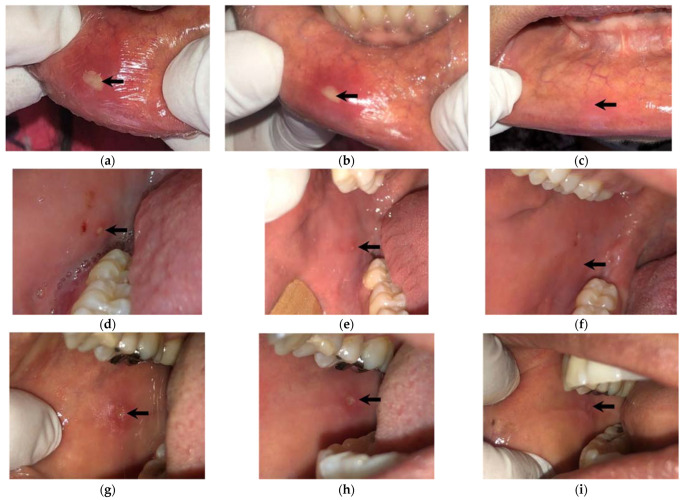
RU in the buccal mucosa, horizontally showing the evolution of the ulcers in three patients; (**a**,**d**,**g**) correspond to the initial state of the RU; (**b**,**e**,**h**) show regeneration after the first application of NTP; finally, (**c**,**f**,**i**) show the healthy tissue 24 h after starting the treatment.

Figure 3 shows, in the usual manner, the evolution of three cases with RU treated with NTP, which resolved within a maximum of 24 h.

**Figure 4:**
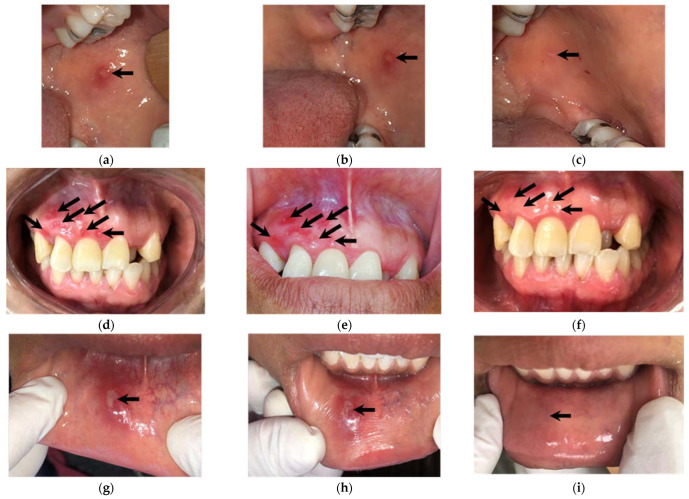
RU is located in different parts of the oral cavity (indicated with arrows): (**a**,**d**,**g**) at patient admission. (**b**,**e**,**h**) after the first application of the NTP. Finally, (**c**,**f**,**i**) show follow-up when the wound has healed (indicated with arrows).

Figure 4 shows three cases of patients with RU who healed on average after three days of NTP application.

## 4. Discussion

Today, no specific drug or treatment is available for recurrent aphthous stomatitis (RAS). RAS healing is a highly specialized process for repairing damaged/injured tissues through various therapies. Failures in the normal healing process of these ulcers lead to abnormal scar formation and a chronic state that is more susceptible to infection. Wounds caused by RU affect patients’ quality of life, which is why there is an urgent demand for the development of competitive therapies. There are new developments in advanced technologies for the care of RU, for example, laser technology [40,41,42], nano-therapy [43,44], etc. Those studies can improve therapeutic results by focusing on tissue regeneration with minimal side effects. Reports show that evolution time and recurrence periods have been reduced [45,46]. The pain caused due to the ulcers experienced by patients with RAS turns out to be the main problem and usually is treated with antihistaminic agents, analgesics, or even anesthetics to reduce pain and promote patients’ oral function [18]. Recent research suggests that ROS/RNS are central players in the actions of antimicrobial and antiparasitic drugs, cancer therapies, wound healing therapies and treatments involving the cardiovascular system. Understanding how ROS/RNS act in established therapies may help guide future efforts in exploiting novel non-thermal plasma-based medical therapies [47,48].

NTP at atmospheric pressure was applied to the ulcer in the cheek, labial mucosa, tongue, oropharynx, mouth floor, soft palate and gingiva. In, patients with RAS and treated with NTP, its anti-inflammatory and healing effect is evident since ulcer size and pain are significantly reduced. A notable improvement was observed since 86% of the patients did not report pain after one hour of application, being statistically significant (*p* = 0.0001). Additionally, at 24 h, 90% of the cases reported being asymptomatic, 7% reported pain with a value of 1 and 3% (one case) reported pain with a value of 4. In this regard, it is essential to point out that the two cases that reported pain with a 1 value correspond to a patient with a significant RAS whose ulcers measured 15 and 19 mm. The case that reported pain with a value of 4 is a smoker and the only case that took seven days to heal; therefore, it is crucial to advise smoking patients with RAS to stop smoking during treatment.

With an average time to repair of three days, considering that more than 50% of the cases healed within two days, it was observed that the application of NTP is efficient for RU management. In contrast, studies using laser treatments reported ten days as a time of recovery [20,49] and others 15 days [50]. Some authors report a decrease in pain and erythema after three days of RU laser treatment; in 75% of cases, they have identified total re-epithelialization five days after beginning treatment [51,52]. However, with these results using lasers, there are still three to five days of epithelial exposure, pain and decreased function remaining for RAS patients. Another of the most widely used treatments for RU is topical corticosteroids, which take five to six days to heal a mouth ulcer [45,53].

Regarding the use of these medications, it is essential to consider the adverse effects that can develop as candidiasis. In addition to treatments based on biological products, they have recently been used for wound healing, reducing pain and recurrence frequency in patients with RAS [54,55]. The NTP application highlights that no drug was applied as an adjuvant.

The initial size of the RU showed a correlation with the time elapsed for tissue repair. That is, the larger the initial size of the ulcer, the longer the repair took, which is explained by the amount of epithelium that needs to be formed. Regarding the type of RAS, it is suggested to repeat the treatment with two applications of NTP at 24 h because the significant RU and herpetiform took four to six days to heal completely.

The findings of this study demonstrated the absence of adverse reactions in the tissues. That is, managing recurrent ulcers with NTP was safe for all treated patients since no case showed adverse effects at six months of follow-up and there was significantly reduced pain levels and repair time after the application of NTP. This could be explained due to the mechanism of action of NTP. Most authors agree that treatment with NTP accelerates wound healing by decreasing the level of inflammatory factors and bacterial load and has anti-neoplastic effects [56]. Reactive oxygen and nitrogen species (RONS) generated by NTP can stimulate blood circulation, causing more significant cellular activity. Thus, restoration potentials are improved by accelerating tissue repair and promoting accelerated tissue regeneration. Several studies demonstrate that the role of RONS, particularly H_2_O_2_, superoxide (O_2_^−^) and nitric oxide (NO), is essential in wound healing beyond the simple oxidative elimination of pathogens. The importance of these elements lies in infection prevention, the induction of angiogenesis, the keratinocyte’s increase in differentiation and migration, and the increase in the proliferation of fibroblasts and collagen synthesis. Furthermore, NO has been demonstrated to participate in an intercellular communication network during the healing process, regulating the behavior of macrophages, keratinocytes and fibroblasts [57]. That is, tissue repair and regeneration steps are highly influenced by these intracellular RONS. Therefore, the application of NTP can be considered to cure RU.

## 5. Conclusions

The study showed that the application of non-thermal plasma in recurrent aphthous stomatitis reduces the levels of pain sensation in the patient, accelerates the healing of the RU and does not produce adverse effects. The technology allowed a quick recovery of the patient and, consequently, a reactivation of daily life. The NTP application in the RUs enables its repair. NTP is an excellent therapeutic alternative for RUs that afflict patients since it provides rapid control of pain and inflammation, accelerating the natural healing process. Due to its application characteristic, it does not bother patients.

## Figures and Tables

**Table 1:** Sociodemographic and risk factors for injuries according to the type of ulcer in this study.

	Recurrent Herpetiform Ulcers	Major Recurrent Ulcers	Minor Recurrent Ulcers	Fisher’s Exact
Gender				
Male			10	0.01
Female	1	10	9	
Age				
18–30		2	9	0.005
31–50	1		7	
51–67		8	3	
Marital status				
Married	1	8	6	0.02
Single		2	13	
Scholarship				
High school		2	3	0.04
Basic	1	8	6	
Bachelor’s degree			10	
Occupation				
Unemployed		3	1	0.003
Student		2	3	
Homework	1	5	1	
Employee			14	
Medication consumption				
Not	1	2	11	0.08
Yes		8	8	
Smoking				
Cigarette		3	4	0.73
No	1	7	15	
Alcohol consumption				
2 to 4 times a month		1		0.02
Never	1	8	4	
Once a year		1	8	
Once a month			7	
Ulcers evolution time				
Two to five days	1	4	10	0.69
Six to ten days		6	8	
One day			1	
Ulcers recurrence period				
Zero			1	0.07
One for year	1	5	2	
One for month		5	5	
One for week			3	
More than a year			8	

**Table 2:** Size, pain and erythema in the initial period and after plasma application, one hour, one day and one week.

Mean and Standard Deviation of Ulcer Size		
Initial	6.9 ± 4.51 A	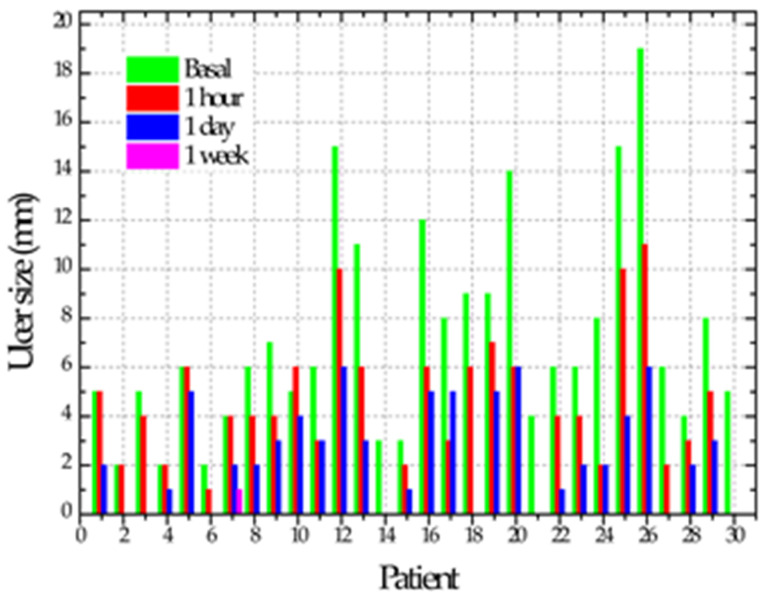
One hour	3.96 ± 3.1 B	
One day	2 ± 2 BC	
One week	0.03 ± 0.18 C	
*p*	<0.0001	
**Mean and Standard Deviation of Pain**		
Initial	7.1 ± 2.5 A	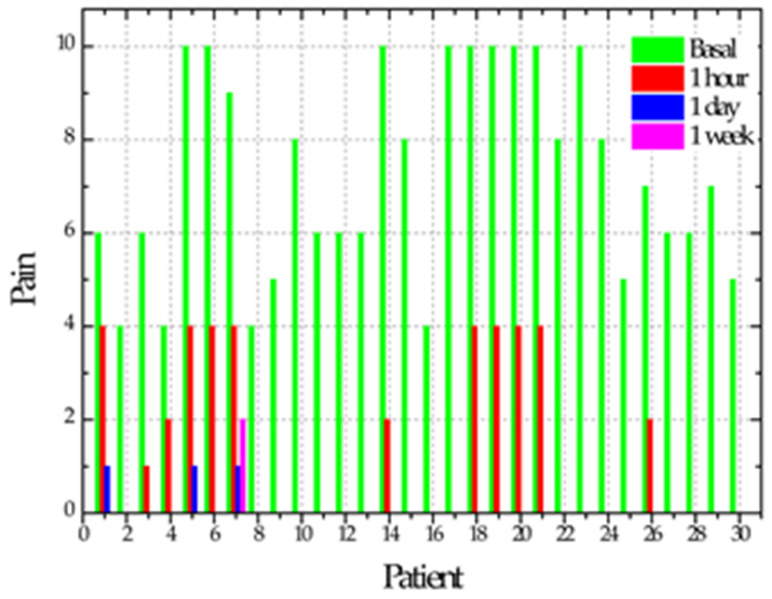
One hour	1.5 ± 2.3 B	
One day	0.2 ± 0.7 C	
One week	0.06 ± 0.3 C	
*p*	<0.0001	
**Mean and Standard Deviation of** **Erythema Size**		
Initial	2.1 ± 1.1 A	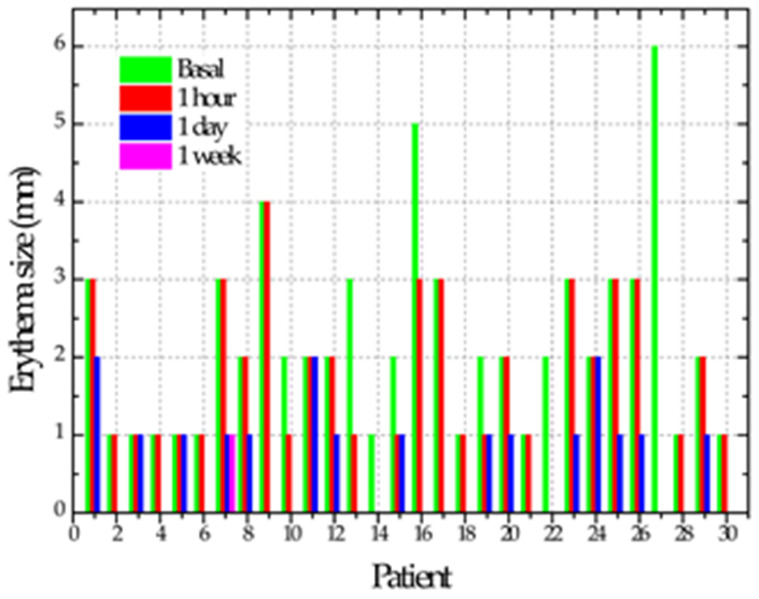
One hour	1.4 ± 1 B	
One day	0.5 ± 0.6 C	
One week	0.03 ± 0.18 C	
*p*	<0.0001	

Different letters = statistically significant differences. It was verified that, after one hour of plasma application, there was a substantial reduction in size and pain (ANOVA one-way test followed by Tukey).

## Data Availability

Not applicable.
